# Considerations on the Condition of Four Mutilés of the Face Home from Germany

**Published:** 1919

**Authors:** 


**Considerations on the Condition of Four Mutilés of the Face Home from Germany.** By Dr. PONT. *La Restauration Maxillo-faciale,* January, 1918.

The author summarizes briefly the clinical history of four mutilés of the face just sent back from Germany and nursed in the author's ward.

One of these patients remained 18 months in Germany, was operated there as many as 6 times, and was given no prosthetic appliance whatever.

Case No. 2 suffered from a double fracture followed by double pseudarthrosis. A symphysary fragment is quite loose and all mastication is impossible. This man received an apparatus in Heidelberg, but could not keep it on for more than 48 hours. When his wounds were closed, he was sent to a prisoner's camp, where he was fed almost entirely on chocolate received from France.

Case No. 3 suffered from a loss of bony substance fatally followed by pseudarthrosis. Although his condition was very bad he was kept prisoner for 3 years.

Case No. 4 presented a constriction of the jaws with a marked deformity which the author had never dealt with before. This man also remained three years in Germany and was submitted there to quite a series of useless interventions.

